# Single posterior approach for circumferential decompression and anterior reconstruction using cervical trabecular metal mesh cage in patients with metastatic spinal tumour

**DOI:** 10.1186/s12957-015-0685-4

**Published:** 2015-08-27

**Authors:** Yen-Chun Chiu, Shih-Chieh Yang, Yu-Hsien Kao, Yuan-Kun Tu

**Affiliations:** Department of Orthopaedic Surgery, E-Da Hospital, I-Shou University, 1, E-Da Road, Jiau-Shu Tsuen, Yan-Chau Shiang, 824 Kaohsiung City, Taiwan Republic of China

**Keywords:** Transpedicular approach, Spinal tumour, TM mesh cage, Metastasis

## Abstract

**Background:**

The goal of surgical management of metastatic spinal tumours is to remove the tumour mass, restore spinal stability and alignment, and provide a better quality of life. A single posterior transpedicular approach, with circumferential decompression, for anterior reconstruction has been advocated to reduce the risk of complication and morbidity associated with a combined anterior-posterior approach. The purpose of our study was to evaluate the clinical outcomes of patients who underwent a single posterior approach for anterior reconstruction at our institution to determine the feasibility and effectiveness of the approach, including the use of a cervical trabecular metal (TM) mesh cage as a vertebral body replacer. As a secondary aim, we evaluated the effect of accumulated experience with the surgical approach on clinical outcomes.

**Methods:**

Twenty consecutive cases of single posterior approach were identified from a retrospective review of spinal surgeries performed at our institution between January 2009 and December 2012. Information on the following clinical outcomes was retrieved from the medical charts for analysis: visual analogue pain score (VAS); neurological status, classified on the Frankel scale; vertebral body reconstruction; spinal alignment, using Cobb’s angle; operative time; volume of blood loss; complications; and the modified Brodsky criteria score, which was used to classify functional recovery as excellent, good, fair, or poor.

**Results:**

Pre- to post-surgical evaluation of outcomes demonstrated a significant decrease in pain (*p* < 0.001), improved spinal alignment, with a mean correction angle of 12° (range, 3°–29°), and higher Frankel score (*p* < 0.001). No severe complications were identified, including deep surgical infection or neurologic deterioration. Eighteen patients achieved good to excellent outcomes, based on the modified Brodsky criteria (*p* < 0.001), with two patients dying within 9 and 11 months of their surgery. Accumulated surgical experience reduced operative time and intraoperative blood loss (*p* ≤ 0.007).

**Conclusions:**

A single posterior approach provided good to excellent clinical and functional outcomes. Based on this evidence, we propose that a posterior approach provides a feasible alternative to the combined posterior-anterior approach for managing patients with metastatic spinal tumours.

## Background

As a result of our aging general population, combined with advances in medical diagnosis and care, the incidence of metastatic spinal tumours has increased significantly over the last decade. Primary spinal tumours are relatively rare, comprising less than 5 % of all spinal tumours. Therefore, spinal tumours are normally metastatic in nature, with the most common sites of primary cancer being the lung, prostate, breast, kidney, and gastrointestinal system. The incidence of secondary spinal tumours is high, with 30–80 % of patients who die of cancer having evidence of spinal metastases on autopsy [1, 2]. The vertebral body is the most frequent site of metastasis, with the intervertebral disc spaces usually being spared.

Surgical treatment for vertebral tumours is commonly assessed through an anterior transthoracic or retroperitoneal approach. Using the anterior approach, the spinal tumour can be removed and direct decompression achieved. After comprehensive debridement and neurological decompression, anterior reconstruction can be performed with an autograft, an allograft, or the use of a body spacer. Supplemental transpedicle screw fixation can be carried out through a posterior approach to provide immediate stability. Therefore, a combined anterior and posterior approach provides the most secure method for the surgical management of metastatic spinal tumours associated with severe destruction of the vertebral body, gross deformity of spinal alignment, and neurological deficits resulting from compression or irritation of neural elements [3–5]. However, most patients with metastatic spinal tumours are elderly and have a poor health status, and therefore, an anterior approach is not recommended due to poor pulmonary function, concurrent medical illness, severe obesity, previous surgery, or previous radiation therapy. Besides, the disadvantages of the combined approach, including the requirement of a long and demanding surgical session and the need to perform a diaphragm takedown or rib-cutting, which may tend to hurt this fragile population, render it an unreasonable procedure.

In an attempt to decrease the morbidity associated with the combined anterior-posterior surgical procedure, a single technique using a posterolateral transpedicular approach (TPA), with circumferential decompression, and anterior reconstruction, has been proposed [6–9]. The application of the TPA technique has been described for the treatment of metastatic spinal tumours, spinal infection, and burst fractures, with an overall report of satisfactory outcomes [6, 10–12]. The purpose of our retrospective, case series study was to evaluate the clinical outcomes of patients who underwent a single posterior approach for anterior reconstruction at our institution to determine the feasibility and effectiveness of the approach, including the use of a cervical trabecular metal (TM) mesh cage as a vertebral body replacer. As a secondary aim, we evaluated the effect of accumulated experience with the surgical approach on clinical outcomes.

## Methods

### Statement of ethics

The experimental design and methods were approved by our institutional review board (EMRP-103-013).

### Participants

Prospective participants were 132 consecutive patients who underwent surgical treatment for metastatic spinal tumours at our institution, from January 2009 to December 2012. Medical records of these prospective participants were reviewed, and 20 patients, who underwent a single posterior surgery with circumferential decompression and anterior reconstruction, using the cervical TM mesh cage, were retained for analysis (Table 1). The study group consisted of 13 women and 7 men, with an average age of 64.5 years (range, 39 to 79 years). Patients’ medical records were reviewed, including outpatient and emergency room notes, admission notes, inpatient progress and nursing notes, discharge summaries, procedure notes, surgical reports, radiology reports, and pathology reports.

**Table 1:** Patient demographic data

Case	Age (yrs)	Gender	Tumour level	Tumour pathology	Instrumentation level	Length of VBR (mm)
1	55	F	L2	Breast ca	T12L1 to L3L4	35
2	72	F	T12	Unknown primary	T10T11 to L2L3	32
3	79	F	L4	Colon ca	L2L3 to L5S1	41
4	74	M	T8	Prostate ca	T6T7 to T9T10	29
5	49	F	T7-9	Myeloma	T5T6 to T10T11	56
6	39	F	L4	Breast ca	L2L3-L5S1	38
7	75	M	L1	Prostate ca	T11T12 to L2L3	41
8	63	F	L1 and L2	Breast ca	T11T12 to L3L4	59
9	65	M	T12	Hepatic ca	T10T11 to L1L2	35
10	57	F	L2	Breast ca	T12L1 to L3L4	32
11	66	M	L3	Rectal ca	L1L2 to L4L5	50
12	56	F	L1	Lung ca	T11T12 to L2L3	32
13	77	M	T11	Unknown primary	T9T10 to L1L2	32
14	62	F	L3 and L4	Bladder ca	L1L2 to L5S1	59
15	77	F	T10	Unknown primary	T8T9 to T11T12	32
16	70	M	L3	Colon ca	L1L2 to L4L5	47
17	76	M	T3-5	Prostate ca	T1T2 to T6T7	62
18	50	F	L1	Hepatic ca	T11T12 to L2L3	35
19	71	F	L2 and L3	Lymphoma	T12L1 to L4L5	62
20	57	F	L4	Breast ca	L2L3 to L5S1	35

*F* female, *M* male, *L* lumbar spine, *T* thoracic spine, *VBR* vertebral body replacement

**Fig. 1:**
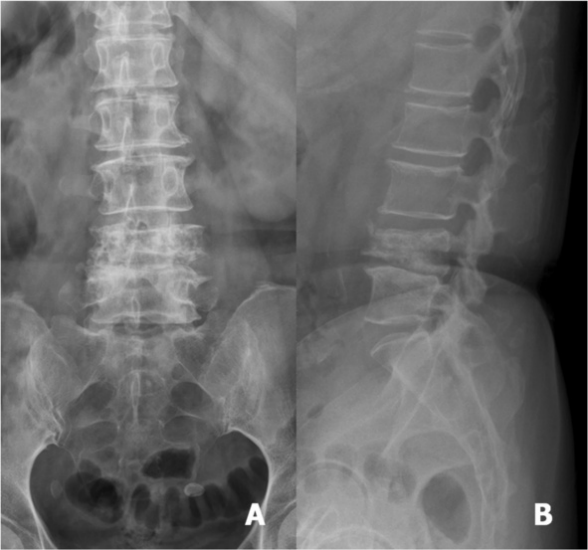
A 57-year-old woman had breast cancer with L4 metastasis. Anteroposterior (**a**) and lateral radiograph (**b**) revealed a collapse of the vertebral body of L4

**Fig. 2:**
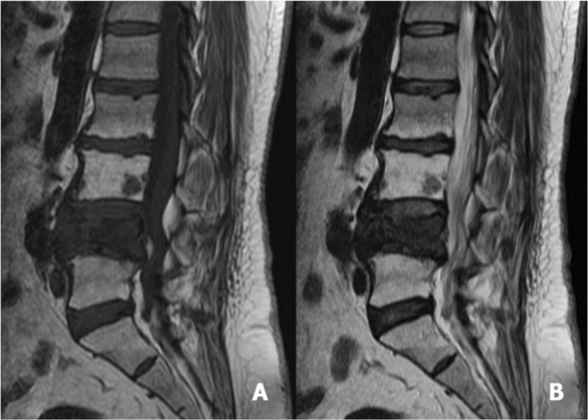
Sagittal T1-weighted (**a**) and T2-weighted MRI (**b**) revealed a pathological fracture of L4, with bone marrow oedema and protrusion into the spinal canal. A small lesion at the body of L3, without a significant effect on structural stability, was identified

**Fig. 3:**
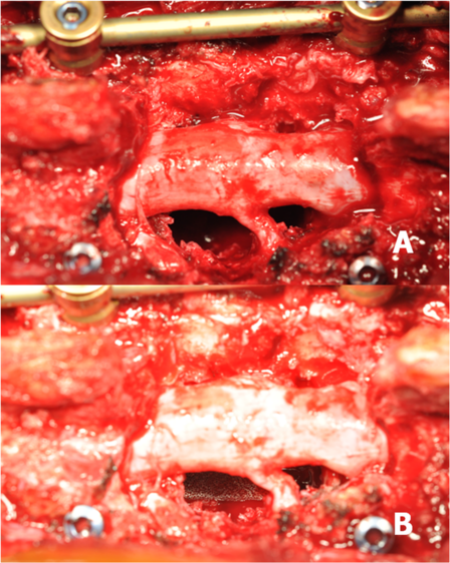
After adequate debridement through a transpedicular approach, the metastatic tumour was identified and removed. A rod was temporarily placed on the contralateral side of the planned TM mesh cage insertion to prevent undesired vibration during operation (**a**). After gently retracting the rod, a well-prepared cervical TM mesh cage was carefully inserted through the route between the nerve roots without sacrifice (**b**)

**Fig. 4:**
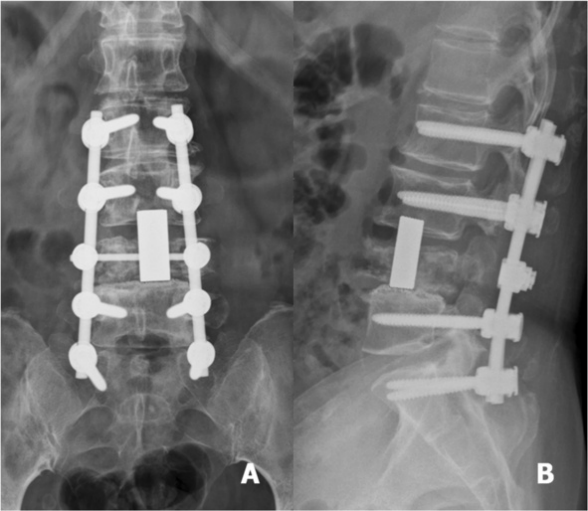
Post-operative anteroposterior (**a**) and lateral (**b**) radiographs revealed that the vertebral body and tumour were removed. Good correction of spinal alignment was achieved by cervical TM mesh cage implantation and posterior pedicle screws fixation

**Fig. 5:**
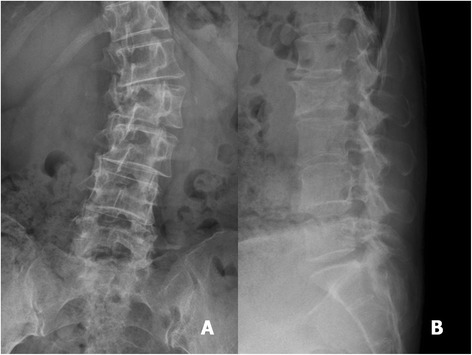
A 55-year-old woman suffered from progressive back pain with bilateral lower extremities weakness. The anteroposterior (**a**) and lateral (**b**) radiographs revealed collapse of the vertebral body of L2 with lateral segmental deviation and malalignment

**Fig. 6:**
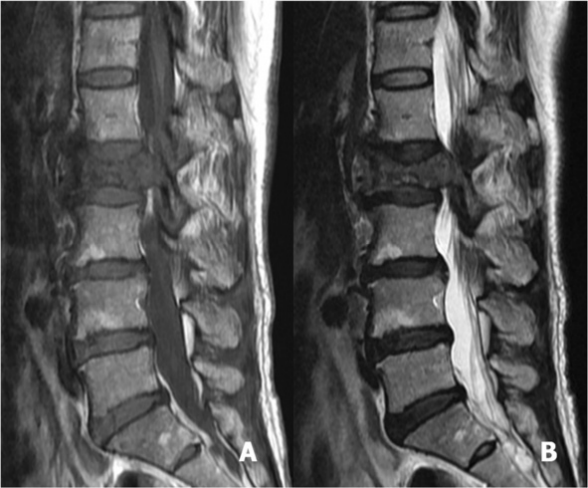
Sagittal T1-weighted MRI (**a**) and sagittal T2-weighted MRI (**b**) revealed a pathological fracture at L2, with spinal cord compression

**Fig. 7:**
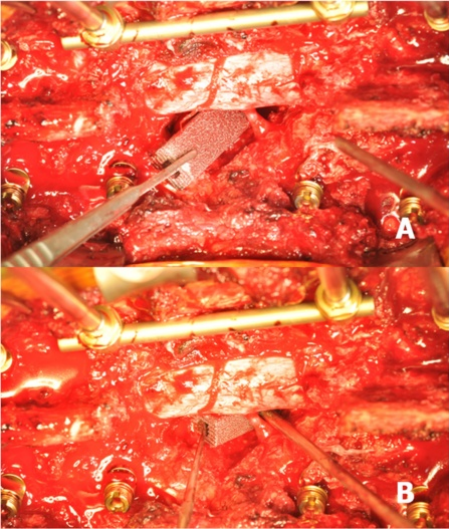
Circumferential decompression through a transpedicular approach was performed (**a**). The cervical TM mesh cage was inserted through the space between the nerve roots without difficulty (**b**)

**Fig. 8:**
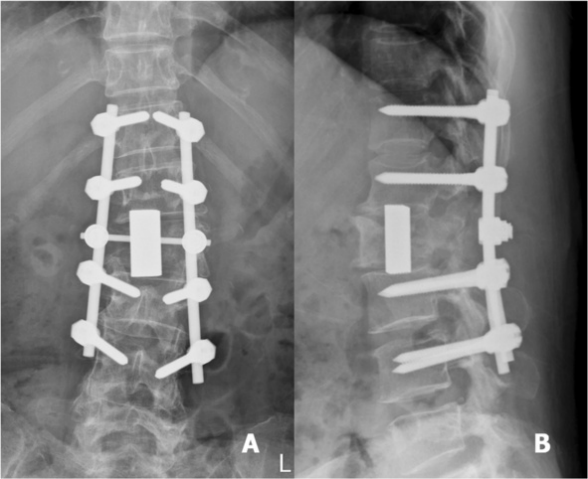
Post-operative anteroposterior (**a**) and lateral (**b**) radiographs revealed an acceptable spinal alignment that was restored with an adequate length of cervical TM mesh cage implantation and posterior pedicle screw fixation

The origin of the metastatic spinal tumours in the 20 patients forming the study group included the breast, colon, prostate, myeloma, liver, rectum, lung, bladder, and lymphoma. All patients shared a common history of progressive back pain that could not be controlled by conservative treatment, including pain medication and use of a brace to provide external support to the spine. For all patients, surgical management was recommended after failure of the conservative treatment (Figs. 1, 2, 3, 4, 5, 6, 7, and 8).

Radiographic assessment of the spine was performed prior to and post-surgery, as well as at each follow-up visit post-discharge, with follow-up being provided through our outpatient department at 1 week, 1 month, and 3 months post-surgeryand at every 3 months thereafter. Two patients died at 9 and 11 months post-surgery, with the remaining 18 patients receiving ≥1 year of follow-up.

### Surgical technique

#### Exposure

Under general anaesthesia, patients were placed in the prone position on foam pads, with the spine protected against further injury and the face, trunk, and extremities positioned to avoid the development of pressure sores. A midline incision was performed, extending two levels above and below the target vertebrae, to expose the posterior complex of the spine. Gentle dissection and meticulous haemostasis was performed to avoid iatrogenic injury to the osteoporotic spine. Intraoperative C-arm fluoroscopy was used to identify the exact vertebral levels for debridement and reconstruction, with the level of exposure adjusted as needed.

#### Posterior stabilization

Transpedicular screws were introduced two levels above and below the involved vertebrae initially. The posterior bone work was initiated by removing the spinous processes with a rongeur. Then, the laminectomy procedure was started and one rod was applied temporarily if spinal instability was found after removing the posterior complex. Since a bulky rod could influence access for cervical TM mesh cage insertion and transpedicular debridement, the temporary rod was placed so as to conduct the screws on the contralateral side of the planned cage insertion. The placement of one rod could maintain adequate stability after extensive posterior complex decompression and prevent injury to neural elements by undesired manipulated vibrations. The planned insertion site was decided based on the pre-operative image survey and neurologic findings. Sometimes, bilateral transpedicular debridement was indicated for extensive debridement. In this situation, we would use another rod on the other side, then release the previously fixed rod after finishing debridement on one side. In this way, decompression of both sides could be achieved.

#### Decompression

Bilateral laminectomy and facetectomy were performed, above and below the involved vertebrae, to expose the involved pedicles and neurological elements. The circumferential debridement procedure was achieved using the transpedicular approach through the interval between the nerve roots. The unilateral approach was used if only one side of the vertebral body was involved, and the bilateral approach was used if both sides were involved. Ligation and sacrifice of the nerve root were not necessary because the working space was adequate. The spinal tumour was removed as completely as possible through the transpedicular route. The discs above and below the involved vertebrae were removed to prepare the adjacent endplates. The anterior structures, either the anterior cortex or the anterior longitudinal ligament, which provided protection for the anterior vessels, diaphragm, visceral organs, and some vital structures, were preserved in most cases.

#### Anterior column reconstruction

After the neural elements were completely decompressed and the tumour mass removed, we gently distracted the two screws above and below the involved vertebrae using a distractor on the rod. During distraction, we needed to observe the nerve roots and spinal cord tension to prevent traction injury to the neurologic elements. The length of the bony defect was then measured. An adequate length of body spacer was prepared and procured. In our cases, we used the cervical TM mesh cage (Zimmer spine, VBR II, Trabecular Metal, 11 mm × 14 mm × height, USA) with length ranging from 29 to 62 mm as reconstruction material. The cylindrical shape with its relatively smaller size on both distal end contact surfaces makes it easier to pass the cage though the space between the nerve roots without scarification. After the cage was well deposited, we released the distraction on the rod and then applied both rods and one link for immediate stability. Sometimes, we even compressed the adjacent instrumentation to obtain good contact between endplate and cage and achieve better spinal alignment.

### Post-operative care

Post-operatively, regular aseptic wound care and pain control were initiated, including a 2-day course of prophylactic antibiotic therapy, with regular assessment of patients’ neurological and hemodynamic status. A Taylor brace or body jacket was applied to protect the spine during early mobilization, initiated on post-operative day 1, as indicated by a patient’s pain and neurological status. All 20 patients were discharged within 14 days of their surgery.

### Outcome assessment

The clinical outcomes were assessed by asking the patients to qualify their pain on a visual analogue scale (VAS, using a scale of 0–10; 0 meaning no pain and 10 the most pain possible) and by evaluating on the basis of pain, their activity, and analgesics requirement during admission, before discharge, and at each follow-up to determine the modified Brodsky’s criteria, which were categorized as poor, fair, good, and excellent. The severity of the neurological status was evaluated using the Frankel scale before surgery, at discharge, and at each visit. The correction of the sagittal Cobb’s angle before surgery was compared with that at discharge using radiographic examination images. The Cobb’s angle, defined as the angle between the superior endplate of the cephadal instrumented vertebrae and the inferior endplate of the caudal instrumented vertebrae, was measured on plain lateral radiographs. Minus (−) indicates the kyphotic angle opposite the lordotic angle.

### Statistical analysis

Data are presented as mean ± standard deviation or median and range, as appropriate for the distribution of the dataset. Clinical outcomes and radiographic findings were compared pre- and post-surgery using the Wilcoxon signed rank test. In order to assess the effect of accumulated experience on surgical outcomes, a sub-analysis was conducted comparing selected outcomes between the group of the first 10 patients undergoing the single posterior approach and the subsequent 10 patients; between-group differences in intraoperative blood loss, operative time, and patient satisfaction were compared using the Mann-Whitney *U* test. A value of *p* < 0.05 was considered statistically significant. All analyses were performed using SPSS 18.0 software (SPSS Inc., Chicago, IL).

## Results

Within our study group, a single posterior approach was used for anterior reconstruction of three spinal levels (*n* = 2), two spinal levels (*n* = 3), and one spinal level (*n* = 15). Instrumentation was implanted from two levels above and below the target vertebrae in 18 patients, with 2 patient requiring extended-level instrumentation due to a severely osteoporotic spine (Table 1). The average length of the TM mesh cage used for anterior reconstruction was 42.2 mm (range, 32–62 mm).

The most prominent clinical sign of metastatic spinal tumours in our patient group was severe back pain, with VAS scores decreasing from 8.2 (range, 7 to 9) before surgery to 3.2 (range, 2 to 4) post-surgery. The modified Brodsky criteria significantly increased for all patients, post-surgery (*p* < 0.001). Of the 20 patients in the study group, neurological function improved significantly post-surgery (*p* < 0.001), with only 4 presenting abnormal Frankel scores of neurological function at discharge. None of the patients in our case series presented deterioration in neurological function post-surgery (Table 2).

**Table 2:** Comparison of visual analogue scale, Frankel scale, modified Brodsky criteria, and Cobb’s angle before and after surgery

Case number	Pre-op VAS	Post-op VAS	Pre-op MBC	Post-op MBC	Pre-op FS	Post-op FS	Pre-op CA	Post-op CA
1	8	2	P	G	C	E	6	14
2	7	3	F	G	D	E	−21	−3
3	8	3	F	G	D	E	22	27
4	8	4	P	G	D	E	−23	−14
5	9	4	P	G	C	D	−56	−41
6	8	3	F	E	E	E	17	20
7	8	3	P	G	D	E	−19	1
8	8	4	P	F	D	D	−5	5
9	8	3	P	G	D	E	−10	2
10	9	3	F	G	E	E	19	31
11	8	3	P	G	D	E	11	16
12	7	3	F	G	D	E	−4	7
13	8	3	P	F	D	D	−15	−9
14	9	4	P	G	D	E	18	34
15	8	3	F	E	D	E	−23	−12
16	8	3	P	G	D	E	20	28
17	9	3	P	G	C	D	−38	−32
18	8	4	F	G	E	E	−12	3
19	9	3	P	G	D	E	−22	7
20	8	2	F	E	D	E	14	33

*Pre-op* pre-operative, *Post-op* post-operative, *VAS* visual analogue scale (0 means no pain and 10 the most pain possible), *MBC* modified Brodsky criteria (*P* poor, *F* fair, *G* good, *E* excellent), *FS* Frankel scale (*A* complete paralysis, *B* sensory function only below the injury level, *C* incomplete motor function below the injury level, *D* fair to good motor function below the injury level, *E* normal function), *CA* Cobb’s angle

The sagittal Cobb’s angle improved significantly, from a mean angle of −6.1° (range, −56°–22°) pre-operatively to 5.9° (range, −41°–34°) post-operatively (*p* < 0.001), with an average angle of correction of the kyphotic deformity of 12° (range, 3°–29°) immediately post-surgery (Table 3). There was no incidence of TM cage dislodgement or loosening of transpedicular screws, as well as no reported incidences of severe surgery-related complications including cerebrospinal liquid leakage, wound infection, pseudoarthrosis, non-union, or loss of fixation, either over the early recovery phase post-surgery or through to the 12-month follow-up. Two patients died due to tumour progression at 9 and 11 months post-surgery.

**Table 3:** Comparison of clinical outcomes and radiographic findings before and after surgery

	Pre-op status	Post-op status	P value
VAS	8.2 ± 0.6^a^	3.2 ± 0.6^a^	<0.001
MBC	P^b^	G^b^	<0.001
FS	D^b^	E^b^	<0.001
CA	−6.1 ± 21.7^a^	5.9 ± 20.7^a^	<0.001

*Pre-op* pre-operative, *Post-op* post-operative, *VAS* visual analogue scale (0 means no pain and 10 the most pain possible), *MBC* modified Brodsky criteria (*P* poor, *F* fair, *G* good, *E* excellent) *FS* Frankel scale (*A* complete paralysis, *B* sensory function only below the injury level, *C* incomplete motor function below the injury level, *D* fair to good motor function below the injury level, *E* normal function), *CA* Cobb’s angle (minus indicates sagittal kyphotic angle (opposed to sagittal lordotic angle))

^a^mean ± standard deviation

^b^median

With regard to our sub-analysis evaluating the effects of accumulated surgical experience, average intraoperative blood loss and mean operative time decreased in the second group of 10 patients, with an average blood loss and mean operative time of 1575 ml and 300.5 min for the first group of 10 patients compared to 1045 ml and 230.5 min for the second group of 10 patients (*p* = 0.007 and *p* = 0.005 for blood loss and operative time, respectively). No other between-group differences were identified in terms of clinical outcomes, based on the MacNab criteria (*p* = 0.654, Table 4).

**Table 4:** Comparison of initial 10 patients and last 10 patients in intraoperative blood loss, operative time, and patient satisfaction

Case	Blood loss (ml)	Operative time (min)	Macnab criteria
Initial 10 cases	1575.0 ± 446.7^a^	300.5 ± 41.8^a^	G^b^
Last 10 cases	1045.0 ± 217.9^a^	230.5 ± 47.6^a^	G^b^
*P* value	0.007	0.005	0.654

*F* female, *M* male, *L* lumbar spine, *T* thoracic spine, *VBR* vertebral body replacement

^a^mean ± standard deviation

^b^median

## Discussion

With advances in medical care, the life expectancy of patients with different cancers has increased. The role of surgery in treating spinal tumours is still evolving and has gained importance in recent years. According to current clinical guidelines, radiotherapy is considered to be the first line of treatment for spinal tumours, with surgical intervention usually reserved for cases of radio-resistant tumours, neurological compromise, mechanical instability of the spine, or failure of conservative treatment [13–15]. With advances in spinal surgical techniques and implants, safe and effective decompression of neural elements and structural stability can now be feasibly achieved. Consequently, the role of surgical intervention in the clinical management of patients with spinal tumours is changing. A randomized prospective study provided evidence of better clinical outcomes with surgical management of spinal tumours compared to radiotherapy, with 84 % of surgical patients in this trial maintaining, and 62 % regaining, the ability to ambulate after treatment, compared to 57 and 19 %, respectively, of patients receiving only radiotherapy [16]. A further prospective clinical study of 85 patients indicated that surgical management of spinal metastatic tumours improved quality of life, with a low rate of complications [17].

The goals of surgical treatment for patients with spinal tumours are to remove the tumour mass, perform a comprehensive decompression of neural elements, restore spinal alignment, and provide immediate spinal stabilization. Although different approaches with various implants have been proposed for treating patients with spinal tumours, circumferential instrumented surgery through a combined anterior and posterior approach is recommended to provide excellent decompression and sufficient spinal rigidity. In their in vitro assessment of spinal stabilization, Wilke et al. demonstrated the superiority of a combined anterior-posterior approach, over either a single anterior or posterior approach, for optimal stabilization [5]. In their review of surgical outcomes for 110 patients having undergone surgery for primary and metastatic spinal tumours over a 5-year period at a single institution, Sundaresan et al. reported that 50 % of patients with spinal malignancies required a combined anterior-posterior approach to achieve adequate tumour removal and spinal stabilization [18]. Harms et al. recommended that an anterior-posterior reconstruction be used for all cases of surgical management of spinal tumours [19].

However, there is a concern over the significant risk for morbidity associated with an anterior approach, including vascular, visceral, or pulmonary complications [20]. In their case series review of 85 patients treated with an anterolateral transthoracic approach for various lesions of the thoracic and thoracolumbar spine, Börm et al. reported severe complications specifically related to the anterior approach in 4 patients (4.7 %) [21]. Although the risk for severe complication with an anterior approach can be considered to be acceptable, an anterior approach is poorly tolerated by patients with poor pulmonary function as it requires deflation of the lungs. Visocchi et al. proposed the following criteria as contraindications to an anterior (transthoracic) approach: pre-operative partial pressure of oxygen (Po_2_) <60, partial pressure of carbon dioxide (Pco_2_) >45, oxygen (O_2_) saturation <90 %, forced vital capacity (FVC) <1.5 l, forced expiratory volume in 1 s (FEV_1_) <1 l, and FEV_1_/FVC <35 % [22]. Most patients with spinal tumours are elderly and have other comorbidities, such as chronic obstructive pulmonary disease and diabetes mellitus. These coexisting diseases increase the risk of severe complications and of unreliable outcomes for these patients. In addition, for spinal tumours located at the thoracolumbar junction, a diaphragm takedown procedure is usually necessary in the anterior approach, which increases the risk for impairment in pulmonary function post-operatively. In fact, in their clinical study of 51 patients who had undergone thoracotomy surgery for the treatment of thoracic adolescent idiopathic scoliosis, Graham et al. reported a significant decline in pulmonary function 3 months post-surgery. Although pulmonary function recovered to within 94 to 96 % of pre-operative baseline values by the 2-year follow-up in these younger patients [23], the impact of the surgical approach on pulmonary function remains a major concern for fragile patients with metastatic spinal tumours.

The posterolateral transpedicular approach with circumferential reconstruction was developed to treat patients with spinal tumours, spinal infection, and burst fractures, with good clinical outcomes reported [6, 10–12]. In their retrospective review of 50 patients with spinal tumours, Metcalfe et al. provided evidence of the effectiveness of a posterior transpedicular approach in providing sufficient access for circumferential decompression and debridement and safe anterior reconstruction using a titanium cage for stabilization [24]. Similarly, Sasani et al. provide evidence of good clinical outcomes, 24 months post-surgery, in 14 patients who underwent single posterior corpectomy with circumferential reconstruction using expandable-cage placement and transpedicular screws for burst fractures [10]. Based on these outcomes, the single posterior transpedicular approach could provide an alternate choice for spinal tumour surgery, reducing operative time, blood loss, complication rate, and hospitalization compared to the traditional combined anterior-posterior approach.

Anterior column reconstruction can be achieved by using an autograft, an allograft, or a body spacer insertion. Autogenous bone graft seems to be the most reasonable material for reconstruction due to its osteoinductive, osteoconductive, and osteogenic cell properties. However, donor-site morbidity is a major concern. Silber et al. retrospectively evaluated donor-site morbidity in 134 patients who underwent a single-level anterior cervical discectomy and autograft interbody fusion procedure using a bone graft from the iliac crest [25]. After an average follow-up of 48 months, they reported the following rates of persistent impairments related to the donor-site: ambulation, 12.7 %; recreational activities, 11.9 %; work activities, 9.7 %; activities of daily living, 8.2 %; sexual activity, 7.5 %; and household chores, 6.7 %.

In comparison to autogenous bone grafts, allograft bone is harvested from an individual other than the patient receiving the graft. Allograft bone, therefore, eliminates the need to create a harvesting surgical site, as well as the associated post-operative pain, and the added expense of a second operative procedure. The disadvantages of allograft bone include the slight chance of disease transmission and a lower effectiveness of the graft as bone growth cells and proteins are removed during the cleansing and disinfecting process. As well, although the risk of transmission of HIV infection with bone allografts has been reported to be less than one case per 1 million uses, the risk is still high considering the life threatening outcomes of HIV infection. Furthermore, the supply of bone allografts is insufficient in most hospitals.

Trabecular metal (TM) technology is an advanced fixation surface designed for orthopaedic implants. With a high coefficient of friction (0.98), TM provides an excellent initial scratch fit. Also, TM material is up to 80 % porous; this high porosity enhances the potential for bone ingrowth and soft tissue vascularization [26, 27]. In their in vivo comparison of polyetheretherketone (PEEK) and porous tantalum (TM) cervical interbody fusion devices in a goat model, Sarina et al. identified bone growth into and around the TM implant margins to be better than for the PEEK devices [28]. Using a cyclical fatigue loading protocol, Ordway et al. examined the interface between the implant and the vertebral endplate in an attempt to model the subsidence identified in vivo [29]. The TM construct demonstrated comparable axial stability and subsidence to a fibular allograft [29]. For the above-mentioned reasons, TM could provide an alternative material for anterior reconstruction in spine surgery.

Although the thoracic nerve roots can be sacrificed during a transpedicular approach if needed, there is concern regarding post-rhizotomy sequelae, including chronic chest neuralgia. The cervical TM mesh cage is relatively small, and therefore, the limited space between nerve roots is sufficient for insertion of the cage for anterior reconstruction. In fact, in our case series, all neural elements were preserved intact.

In our retrospective case series, we demonstrated that the single posterior transpedicular approach with cervical TM mesh cage reconstruction provides favourable clinical outcomes, even in the upper thoracic spine where the transpedicular space is relatively small. There was no major surgery-related complication, although three patients (15 %) experienced post-operative paraesthesia. The post-operative radiographs identified mild subsidence of the TM mesh cage in eight patients. However, there was no TM mesh cage displacement or dislodgement, no pedicle screw loosening or pullout, and no implant-related undesired clinical symptoms. The complication rate was encouraging compared to the reported morbidity rate of 48 % for the combined anterior and posterior approach [30].

The mean operative time in our case series was 265.5 min (230.5 min for the last 10 cases and 300.5 min for the initial 10 cases), with a mean blood loss of 1310 ml (1045 ml for the last 10 cases and 1575 ml for the initial 10 cases). Both blood loss and operative time decreased significantly with accumulated surgical experience. Although we could not find a significant difference between the initial and last 10 cases in clinical outcome based on MacNab criteria, this does not erase the importance of surgical experience. Most spine surgeons are more familiar with posterior midline and posterolateral approaches for the treatment of degenerative pathologies, and the posterior transpedicular approach represents a relatively straightforward and natural extension of their expertise. Therefore, after a steep learning curve, most spinal surgeons can easily and confidently use the single posterior approach to treat patients with spinal metastatic tumours without worrying about anterior approach-related problems and complications.

Most spine surgeons agree that the major role of surgical treatment in patients with metastatic spinal tumour is improving quality of life. The treatment protocol depends on an individual patient’s overall health, ambulatory status, tumour type, tumour load, spinal stability, and presence of neurologic compromises. Tokuhashi et al. provided a scoring system, which includes six main patient-related characteristics, for pre-operative evaluation of metastatic spine tumour prognosis [31]. They suggested that a predicted prognosis of greater than 6 months is an indication of surgical treatment. All of the patients in our case series lived more than 6 months, and their quality of life improved significantly after surgical treatment.

## Conclusions

Our retrospective case series study provided evidence of the feasibility and effectiveness of a single posterior approach as an alternative method for patients who are not suitable for or cannot tolerate a combined anterior and posterior approach. A cervical TM mesh cage can be considered as an alternative material for anterior reconstruction, eliminating the concern of donor-site morbidity associated with autografts and transmitted diseases with allografts.

## Abbreviations

TM: trabecular metal
VAS: visual analogue score
TPA: transpedicular approach
FVC: forced vital capacity
FEV1: forced expiratory volume in 1 s
PEEK: polyetheretherketone
